# Relationship between Diabetic Retinopathy and Subclinical Hypothyroidism: a meta-analysis

**DOI:** 10.1038/srep12212

**Published:** 2015-07-20

**Authors:** Jingyang Wu, Song Yue, Jin Geng, Limin Liu, Weiping Teng, Lei Liu, Lei Chen

**Affiliations:** 1 Department of Ophthalmology, The First Affiliated Hospital of China Medical University, Shenyang, 110001, China; 2 Department of Endocrinology and Metabolism, Institute of Endocrinology, Liaoning Provincial Key Laboratory of Endocrine Diseases, The First Affiliated Hospital of China Medical University, Shenyang, 110001, China; 3 Department of Epidemiology. School of Public Health, China Medical University, Shenyang 110001, People’s Republic of China

## Abstract

Several epidemiological studies have found a positive association between diabetic retinopathy (DR) and subclinical hypothyroidism (SCH), but the findings are varied or even contradictory. In the work, we performed a meta-analysis to ascertain the relationship between DR and SCH. We searched relevant studies on the relationship between DR and SCH. All English reports were used Medline, EMbase, Web of Science, Google scholar, and all Chinese ones used CBMDisc (Chinese Biochemical Literature on Disc) and CNKI (China National Knowledge Infrastructure) database. Meta-analysis was performed using RevMan 5.1 software. We obtained eight observational studies. Random-effects meta-analysis indicated a significant association between DR and SCH (odds ratio = 2.13, 95% confidence interval = 1.41 – 3.23, p < 0.001). Based on currently evidence, SCH is probably a significant risk factor for DR.

Type 2 diabetes mellitus (T2DM) is becoming increasingly prevalent worldwide. Diabetic retinopathy (DR) is the most common ocular complication of diabetes, and is the leading cause of visual impairment and blindness in working-aged people^1^. DR is common in diabetic patients but is asymptomatic until a significant visual impairment occurs. Late diagnosis of DR results in the socio-economic burden of illness associated with diabetes^2^. According to the World Health Organization (WHO), blindness due to DR accounts for 4.8% of the total thirty-seven million cases of blindness around the world in 2006^3^. Therefore, it is important to investigate the risk factors that promote or predict the onset and development of DR.

Subclinical hypothyroidism (SCH) is a common endocrine disorder and characterized as elevated serum thyroid-stimulating hormone (TSH) levels in the presence of serum free thyroxine (FT4) and triiodothyronine (T3) levels within the reference range^4^. In general population screening surveys, the prevalence of SCH has been reported to range from 4% to 10%^5^, and the risk factors of SCH are baseline TSH level^6^, iodine-sufficient^7^,^8^,^9^, old age, female sex, and the presence of thyroid autoantibodies^10^,^11^,^12^,^13^,^14^.

The association between T2DM and SCH is well known, with the reported prevalence of SCH in diabetes varying between 2.2% to 17%^15^,^16^. Recently, several researches investigate the relationship between SCH and DR, but the results were inconsistent. Kim Bo-Yeon *et al.*^17^ considered that SCH was associated with DR, but Chen *et al.*^18^ took the opposite opinion.

Consequently, we performed this meta-analysis of eligible observational studies in order to more precisely investigate the relationship between SCH and DR.

## Results

**Figure 1:**
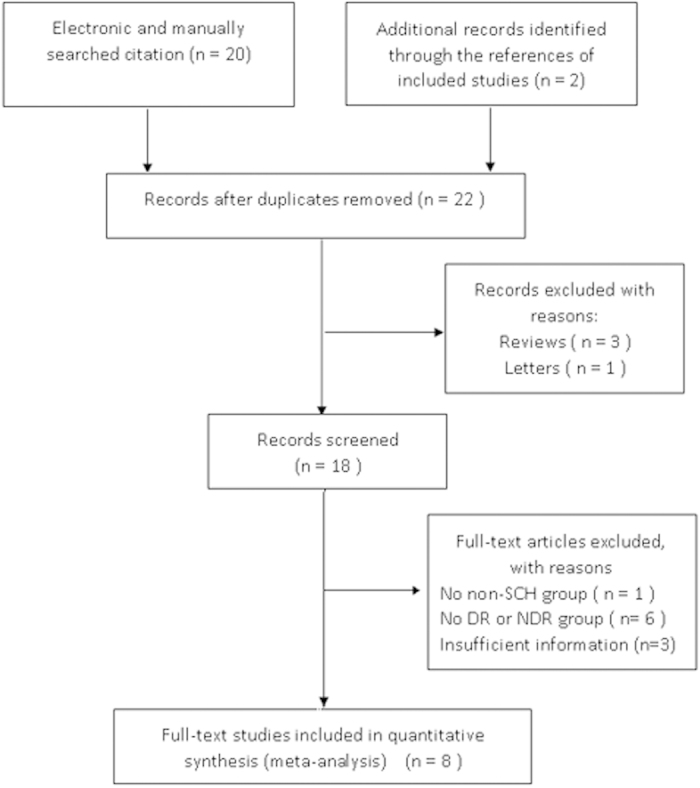
Flow chart demonstrating those studies that were processed for inclusion in the meta-analysis.

We screened 22 articles published from 2010 to 2014. Of these, 14 studies were excluded for the following reasons: reviews, letter and not provide sufficient information for estimating the relationship between DR and SCH. Thus, 8 studies were included in this meta-analysis. Figure 1 was the selection process and reasons for excluding studies.

The major characteristics of the eight studies are shown in Table 1. The included studies were published in Chinese and English, and preformed in Chinese mainland, Taiwan and Korea. All of them were hospital-based case-control studies and SCH were all defined as TSH level >4.0 uIU/mL in the presence of normal serum free thyroxine level (0.7–2.0 ng/dL).

**Figure 2:**
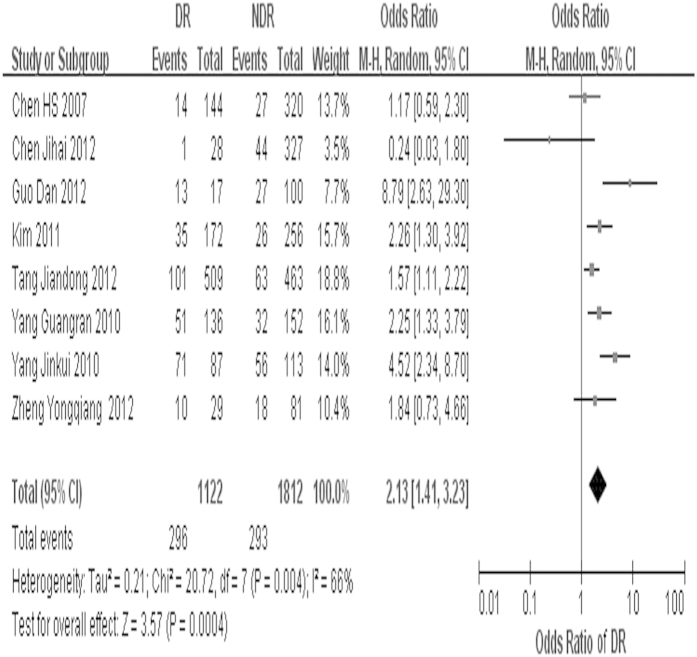
Forest plot of SCH and risk of DR, studies are pooled with random-effects model.

The overall OR estimates for each study were pooled to give a total estimated of risk (Fig. 2). Obviously heterogeneity was observed (p = 0.004, *I*^*2*^ = 66%), so the result based on the random-effects model showed that exposure to SCH can increase the DR risk 2.13 times (OR = 2.13, 95%CI = 1.41 – 3.23, p < 0.001).

**Figure 3:**
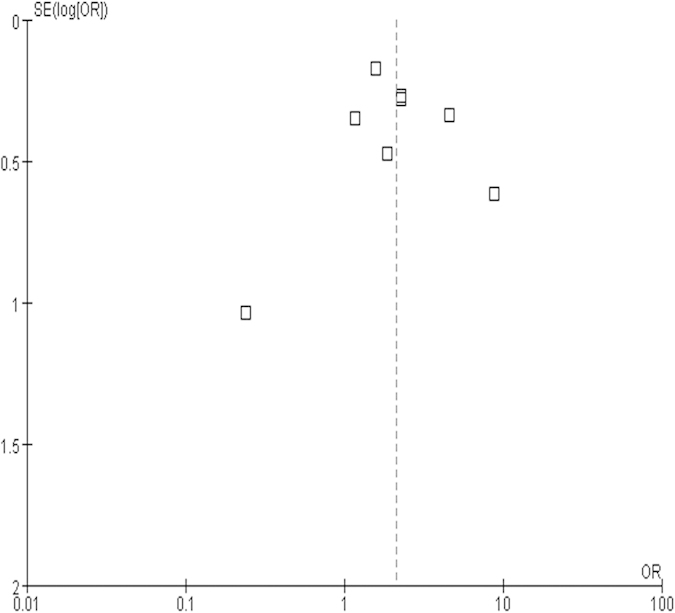
Funnel plot based on 8 case-control studies.

Figure 3 was the funnel plot of the research. The result shows that the figure was not asymmetrical, which demonstrated that there was no significant publication bias.

## Discussion

It is well-known that, DR is one of the most common microvascular complications and the leading cause of blindness worldwide. Recently, as the number of people with T2DM increased, the prevalence of DR is rapidly increasing^19^,^20^. The results of a meta-analysis from the population-based studies around the world showed that the overall age-standardized prevalence of any DR was 34.6%^21^. Because the symptoms of the early stage in DR are not apparent, patients often miss the best opportunity for treatment when diagnosed, which is leading to a high rate of blinding. According to the World Health Organization (WHO), blindness due to DR accounts for 4.8% of the total thirty-seven million cases of blindness around the world^22^. Therefore, it is important to investigate the risk factors for DR. In the past researches, the well-known risk factors for the development of DR include duration of diabetes, poor glycemic control, elevated blood pressure, and dyslipidemia, of which the latter three are potentially amenable to therapeutic intervention^23^,^24^.

In recent years, several investigators discussed the relationship between DR and SCH, but the results were contradictory. Accordingly, this study was aimed to estimate the pooled size of SCH in DR risk by a meta-analysis.

In this research, the pooled effect estimated from eight included papers demonstrated a 2.13 fold (95%CI = 1.41 – 3.23, p < 0.001) increased risk of DR in SCH patients compared with non-SCH individuals. But substantial heterogeneity was observed among the studies. To find out the sources of heterogeneity, we exclude each one study at a time. Heterogeneity was obviously decreased when excluding the following three studies respectively, which indicating that the three studies were the source of heterogeneity. They are studies by Yang Jinkui *et al.*^25^, Chen Jihai *et al.*^26^ and Guo Dan *et al.*^27^. Sample size of study by Guo Dan *et al.* was only 157, that may be the reason for its heterogeneity. Chen Jihai *et al.* only selected over 65 years old people as study objects. So there may be selection bias in that study. Similarly, Yang Jinkui *et al.* randomly selected 127 SCH patients and 200 non-SCH people from 1170 T2DM patients, but the method of selecting was unclear.

Above all, this is the first meta-analysis investigating the association between DR and SCH, and it demonstrated that DM patient suffering from SCH could increase the risk of DR. But it differed from those of Chen *et al.*^18^ and Zheng Yongqiang *et al.*^28^. Reasons for this discrepancy may be related to differences in characteristics of the participants.

Several mechanisms may be involved in the association between DR and SCH.

First of all, insulin resistance. Several studies have found that fasting hyperinsulinemia or insulin resistance (IR) was associated with SCH^29^,^30^. Previous studies indicated that IR was associated with the presence of DR in T2DM^31^,^32^, and the main mechanism was defective fibrinolysis or impaired vasodilation associated with IR^33^,^34^. It may be correlated with the reduction of vasodilation ability and fibrosis caused by IR, which led to destruction of retinal vessel and secondary revascularization^35^,^36^.

Secondly, serum C-reactive protein (CRP). Researches by Christ-Crain *et al.*^37^ and Kvetny *et al.*^38^ indicated that the level of CRP in patients with SCH was obviously higher than that in non-SCH people. The study of Van Hecke *et al.*^39^ shown that there was significant relationship between CRP and DR. As is well known, DR is a Chronic inflammatory disease, which is correlated with the inflammation-mediated injury of vascular endothelial cell. Moreover, CRP is recognized one of the primary and most sensitive acute phase protein in human nonspecific inflammation reaction^40^.

The third, the level of serum homocysteine (Hcy) in SCH patients was much higher than non-SCH people. Hcy is a reactive amino acid about vascular injuries. Looker *et al.*^41^ demonstrated that elevated levels of Hcy was the risk factor for DR. The reason may be that Hcy could enhance the lipid peroxidation^42^, which leads to increased levels of oxidized low density lipoprotein(OX-LDL), accelerating the progresses of vascular disease^43^. Besides, some scholars have found that the mRNA expression of VEGF was significantly enhanced with the concentration enrichment of Hcy^44^. Moreover, it is generally recognized that higher increase of VEGF was significantly associated with DR. Consequently, Hcy may promote the occurrence and development of DR by approaches mentioned above.

The forth, oxidative stress. The activity of paraoxonase 1(PON1) and superoxide dismutase (SOD) in the plasma of SCH patients is significantly lower than that of normal control. That is to say, antioxidative capacity of SCH has dropped significantly. However, there were some literatures have shown that oxidative stress was an important risk factor for promoting the occurrence and development of DR^45^.

The last, correlation between DR and dyslipidemia has been reported^46^, and atherogenic disturbances in lipid metabolism have been observed in patients with SCH^47^,^48^. Thus, dyslipidemia in SCH may be one of the reasons for the association between DR and SCH.

This study demonstrated that SCH could increase the risk of DR, but whether SCH could be a biomarker for DR is also unclear. Therefore, animal experiments should be performed to investigate the correlated mechanism between DR and SCH. The results can give implications for clinical practice and support the link between the two diseases.

All the included studies were performed in Asian countries. Therefore, we suggest the western countries to conduct relevant studies to verify this prediction.

We acknowledge that the current findings are cross-sectional in nature and the availability of prospective data would further improve confidence in these associations.

Although this is the first meta-analysis investigating the association between DR and SCH, which is very important for prevention of DR, there were some limitations. First, all the literature searches were hospital-based studies, absence of population-based studies. Second, as we cannot have access to unpublished results, publication bias cannot be excluded. Third, the literature search was limited to be published in English or Chinese. The last point, the number of studies for our meta-analysis is not large and the sample sizes in some studies were slightly small. Compared with the studies having a large sample size, studies with a small sample size may overestimate the true association. A large sample study with either finding may better reflect a true association because of its sufficient statistical power.

In conclusion, the meta-analysis of all published epidemiological studies on DR and SCR revealed that SCH was associated with DR in diabetes and exposure to SCH can increase the DR risk 2.13 times.

## Methods

### Search Strategy

Our research objects are all human species. We searched all English publications using Medline, Embase, Web of Science, Google scholar, and searched all Chinese publications manually and on-line using CNKI (China National Knowledge Infrastructure) and CBMDisc (Chinese Biochemical Literature on Disc) database. The search keywords were: (1) “diabetic retinopathy” OR “diabetic microangiopathy”; (2) “subclinical hypothyroidism”; (3) “association” OR “relationship”. A total of 22 reports published in the period from 1991 to 2013 were identified.

### Inclusion and Exclusion Criteria

Reports were considered eligible for inclusion if the following criteria were met: (1) full-text could obtain; (2) clear diagnostic criteria for SCH and DR were reported; (3) the adjusted or unadjusted hazard ratios (HRs), relative risks (RRs), or odds ratios (Ors) could obtain, associated 95% confidence intervals (Cis) or the numbers of events that can calculate them were reported. If more than one study covered the same population, only the study of highest quality was included.

Reports were excluded if the following criteria were met: (1) the study without specific sample origins; (2) the study with a sample size less than 50; (3) the data in the study was obviously paradoxical or not present clearly enough. A total of 22 relevant studies were screened. After systematic review, only 8 of these were included in the meta-analysis. The progress for study inclusion is shown in Fig. 1.

### Data Extraction

There were two researchers, Jingyang Wu and Lei Liu, independently reviewing all the potentially relevant papers through assessing the eligibility of each article and abstracting data with standardized data-abstraction forms. Disagreements were resolved through discussion. The characteristics of these studies included in this meta-analysis on the association of DR and SCH are shown in Table 1.

### Data Analysis

Ors and relevant 95% Cis were computed using Review Manager (RevMan, version 5.1, Copenhagen: The Nordic Cochrane Centre, The Cochrane Collaboration, 2011). Ors were used to measure association across the studies. Heterogeneity evaluation of all the studies were used the Chi-square based *Q* test and *I*^*2*^ test^49^. Heterogeneity was assessed with low, moderate, and high *I*^*2*^ values of 25%, 50%, and 75%, respectively with the *I*^*2*^ statistic^50^. If moderate or high level heterogeneity existing, a random-effects meta-analysis was performed, unless using fixed-effects models. Publication bias was assessed by visually inspecting a funnel plot. A p value less than 0.05 was considered statistically significant^51^,^52^.

## Additional Information

**How to cite this article**: Wu, J. *et al.* Relationship between Diabetic Retinopathy and Subclinical Hypothyroidism: a meta-analysis. *Sci. Rep.* **5**, 12212; doi: 10.1038/srep12212 (2015).

## Figures and Tables

**Table 1:** Characteristics of included studies in the meta-analysis.

**First Author**	**Region**	**Study design**	**Publication year**	**Relationship between DR and SCH**	**Sample size**	**Age(years)**	**SCH sample**	**DR sample**
Kim Bo-Yeon^7^	Korea	Case control	2011	YES	489	61.7 ± 9.8	61	207
Chen HS^8^	Taiwan	Case control	2007	NO	588	67.2 ± 10.8	41	158
Yang Jinkui^11^	China	Case control	2010	YES	327	61.0 ± 13.7	127	158
Chen Jihai^12^	China	Case control	2012	YES	400	75.53 ± 6.53	45	29
Guo Dan^13^	China	Case control	2012	YES	162	55.84 ± 13.03	40	30
Zheng Yongqiang^14^	China	Case control	2012	NO	138	60.2 ± 11.8	28	39
Yang Guangran^53^	China	Case control	2010	YES	371	59.94 ± 10.61	83	187
Tang Jiandong^54^	China	Case control	2012	YES	1156	64.6 ± 10.2	164	610
